# Uncertainty and Conservatism in Assessing Environmental Impact under §316(b): Lessons from the Hudson River Case

**DOI:** 10.1100/tsw.2002.198

**Published:** 2002-03-06

**Authors:** John R. Young, William P. Dey

**Affiliations:** ASA Analysis & Communication, State College, PA 16801, USA; ASA Analsyis & Communication, 51 Old State Road, Wappingers Falls, NY 12590, USA

**Keywords:** uncertainty, conservatism, entrainment, impingement, 316(b), power plant impact, environmental impact

## Abstract

Initially, regulation of cooling water intakes under §316(b) was extremely conservative due to the rapid increase predicted for generating capacity, and to the uncertainty associated with our knowledge of the effects of entrainment and impingement. The uncertainty arose from four main sources: estimation of direct plant effects; understanding of population regulatory processes; measurement of population parameters; and predictability of future conditions. Over the last quarter-century, the uncertainty from the first three sources has been substan-tially reduced, and analytical techniques exist to deal with the fourth. In addition, the dire predictions initially made for some water bodies have not been realized, demonstrating that populations can successfully withstand power plant impacts. This reduced uncertainty has resulted in less conservative regulation in some, but not all venues. New York appears to be taking a more conservative approach to cooling water intakes. The conservative approach is not based on regulations, but in a philosophy that power plant mortality is an illegitimate use of the aquatic resources. This philosophy may simplify permitting decisions, but it does not further the development of a science-based definition of adverse environmental impact.

> **Unless steps are taken to find alternate means of dispersing or utilizing this heat, there is a distinct possibility that all major rivers in the United States will reach the boiling point by 1980 and then evaporate entirely by 2010! —** Richard Wagner in *Environment and Man*, 1971[1]

> **By the year 2000 the water flow through the condensers of power plants will exceed two million cubic feet per second, approximately 1.2 times the average freshwater discharge of the 48 contiguous States. —**C.P. Goodyear and B.L. Fodor in Ecological Implications of Anticipated Electric Power Development, 1977[2]

> **The staff analysis indicates that during June and July of most years from 30 to 50% of the striped bass larvae which migrate past Indian Point from upstream spawning areas are likely to be killed by entrainment. .... As a result, there is a high probability that there will be an initial 30 to 50% reduction in the striped bass fishery which depends upon the Hudson for recruitment.** —Atomic Energy Commission, Final Environmental Statement Related to Operation of Indian Point Nuclear Generating Plant Unit No. 2, 1972[3]

Although two of these quotes refer to the discharge of waste heat from power plant cooling systems and the need for cooling water, rather than to direct entrainment and impingement impacts, they nevertheless epitomize the attitude, prevalent at the time §316 was enacted, that once-through cooling systems would create huge environmental problems. These attitudes were fostered not only by a relatively rudimentary knowledge of the actual impacts of once-through cooling, but also by the projections for growth of electrical demand and especially nuclear power as a means of satisfying that demand. Projections were made that by 2000, the nationwide generating capacity would need to be 1,575,000 MW, nearly three times the capacity available in 1976[4].

Given the predictions for increasing electrical demand, the resultant need for cooling water, and the lack of information available on the effects of one-through cooling, it is not surprising that the new United States Environmental Protection Agency (USEPA) would take a conservative regulatory view, i.e., to err on the side of being over-protective regarding the use and discharge of cooling water. However, even in their conservatism, the agency focused on preventing effects at the population and ecosystem level. The guidance manuals provided by the agency clearly were directed at assessing and preventing impacts at the levels of populations and communities[5].

The conservative view to regulation was considered necessary because assessment of the impacts of power plant operations were highly uncertain. The uncertainty arose from four distinct sources. First, the direct effects on aquatic organisms were difficult to measure, and estimates were fraught with numerous untested assumptions. For instance, without any demonstration to the contrary, it seemed prudent to assume that all organisms entrained into the cooling system would be killed[6]. In addition, the calculation tools used to estimate numbers killed or a fraction of the population killed by power plants contained many parameters that were not amenable to empirical description with the data available at the time. Therefore, it was necessary to assess the sensitivity of the results to a range of assumed values for these parameters.

A second component to uncertainty was the incomplete knowledge of the processes that affect the population dynamics of the resident aquatic species. In the 1970s, the large ecological studies of power plant impacts (e.g., Hudson River, Delaware Bay, Niantic River) were just getting started. Many of these studies were conducted on estuarine systems. Although often very productive, estuaries are also highly variable, which makes it difficult, if not impossible, to understand population regulatory processes with only a few years of study. Assessments of impact conducted in the late 1970s typically had less than ten years of data available, therefore the understanding of the factors that influence the population dynamics of affected species was preliminary at best.

Sampling variability adds to the uncertainty in measuring population characteristics and the effects of power plants on these characteristics. Catches of fish in sampling programs are highly variable, thus estimates of abundance often have large confidence bounds. Life histories of many of the affected species are complex, involving only temporary occurrence near the power plants and/or long annual migrations, making them extremely difficult to sample for some parts of the life cycle. Invariably, all fish in a cohort do not follow the same life history pattern. For anadromous species, some individuals emigrate from the estuary at an earlier age than others, and similar variation exists for time and age at return. The length and timing of ocean migrations are also variable, as are growth, maturity, and fecundity.

Finally, uncertainty of future conditions also adds to the imprecision of our ability to predict impacts on future populations. Even if we had perfect knowledge of the direct impacts, the processes that regulate the population, present population characteristics, changes in climatic conditions, current patterns, habitat alterations, and commercial or recreational fishing mortality rates may occur in the future, which would then make our predictions of the future populations uncertain.

The result of these four sources of uncertainty was that regulation under 316(b) was initially very conservative and closed-cycle cooling was frequently mandated as the best available technology. During the 1970s the frequency of use of the various designs of cooling systems for new plants changed radically. For plants that began operating prior to 1970 and plants less than 500 MW prior to 1973, once-through cooling accounted for 75% of installed capacity with closed-cycle cooling comprising only about 10%. For plants completed after 1978, 80% of the capacity was cooled by closed-cycle systems, while once-through cooling was used at less than 5%[7].

Despite the clear trend toward closed-cycle cooling, some plants were able to reach agreement with USEPA and other regulatory agencies and find alternative measures to minimize adverse environmental impact; however, this was not easily accomplished. For example, the 1975 draft NPDES permits for the new Hudson River plants (Indian Point, Bowline Point, and Roseton) all contained conditions that would eliminate once-through cooling and greatly reduce the entrainment and impingement of fish. Finally, after lengthy legal proceedings, a settlement was achieved that reduced potential fish mortality through flow restrictions, appropriately timed outages, intake modifications, and mitigative stocking[8].

The key to reaching agreement on cooling system requirements lies in reducing the uncertainty of the assessment from as many of the four components as possible. In the Hudson River case, one of the key factors was the convergence of the estimates of direct power plant effects that was achieved as the technical experts from both sides met and discussed the impact models[9,10]. Part of this convergence was due to the clear demonstration that mortality of entrained organisms can be considerably less than 100% for particular species and life stages[11,12].

Uncertainty of the underlying ecological processes can also be reduced through long-term monitoring studies that provide a wider range of the conditions that affect the population in various ways and validate the predictions of the earlier methodologies. In the Hudson River, continuation of the environmental studies for nearly 30 years has provided the opportunity to observe both high and low abundance periods for striped bass and other species in response to fishing mortality rates, a wide range of climatic variation, and different levels of power plant mortality[13]. In addition, other human influences on the estuary have also changed dramatically over this time period. Untreated or inadequately treated sewage discharges to the estuary have been largely eliminated, with a concomitant improvement in water quality[14]. Chemical control of the invasive water chestnut (*Trapa natans*) was discontinued, resulting in a tremendous resurgence of the species in the freshwater regions of the estuary. In the early 1990s, zebra mussels (*Driessena polymorpha*) appeared in the freshwater portions of the estuary and caused a substantial alteration of the lower levels of the estuarine food web[15]. Long-term studies afford the opportunity to observe these ecologically important events, which offer unique opportunities for insights to population regulatory mechanisms.

It is impossible for any monitoring program to study all aspects of the environment that may be important in understanding the population dynamics of species subject to entrainment and impingement. It is critical to proper 316(b) evaluation to be aware of and facilitate other research efforts that could provide additional crucial information. In the Hudson River, there has been a great deal of other research conducted through funding provided by the Hudson River Foundation, by the New York Department of Environmental Conservation (NYSDEC) for fishery management purposes, and through other avenues. Through the years the owners of the Hudson River stations have attempted to promote these other research efforts through co-funding of projects, cooperating with researchers in collecting specimens, and by making the utility data available for legitimate research needs. These efforts have succeeded in assisting crucial pieces of scientific research that have helped elucidate some of the possible population regulatory mechanisms[16,17,18,19]. However, it must be remembered that monitoring studies provide no guarantee that they will uncover the primary regulatory processes[20], and will never be able to prove that particular mechanisms are the prime regulatory factors. They can, however, increase the confidence that the true regulatory processes are identified and understood.

Measurement uncertainty can also be reduced substantially with carefully designed and executed sampling programs. These programs need to consider inherent sampling variability and use sufficient sample sizes to provide suitably precise estimates. Data from the Hudson studies were used to determine how sample size and precision are related[21], knowledge which can be used to design an effective sampling program.

The always imperfect knowledge of future conditions may also be addressed in various ways. In choosing fisheries' harvest policies, the uncertainty is often ignored without substantially affecting the performance of the fishery; however, when mortality is high enough to permanently alter the health of the stock, explicit adjustment of policies for the uncertainty is preferable[22]. Explicit inclusion of uncertainty can be done through risk analysis if probabilities can be assigned to various possible future states[23,24]. Other techniques, such as fuzzy math[25], sensitivity analyses[26], and meta-analysis[27], can be used when information on probabilities is not available.

In some areas, fisheries management is moving toward the "precautionary approach" to setting management controls[28,29], and this approach may also be useful for 316(b) regulation. The precautionary approach explicitly recognizes the uncertainty of biological information and the imperfect ability of management policies to assure that biological targets are met. In recognition of this uncertainty, targets are set in a conservative manner so that the probability that numerical biological reference points, such as the minimum acceptable spawning stock biomass, are exceeded is acceptably low. The level of conservatism of the management policies varies directly with the level of uncertainty.

As a result of all the research and monitoring conducted since 316(b) was enacted, our understanding of the effects of entrainment and impingement in 2001, while still imperfect, is far better and less uncertain than it was in 1972. However, given that some uncertainty is still present, some will argue that conservative regulation, erring on the side of over-protection of aquatic species, is still the best policy for 316(b). If over-protection came at no cost, without trade-offs among other socially and ecologically beneficial attributes, then it would be difficult to argue against this position. After all, the technology exists to practically eliminate fish entrainment and impingement by using closed-cycle cooling. Unfortunately there are trade-offs to be made, and it is prudent to examine these trade-offs before settling on a final position on uncertainty and conservatism.

One of the trade-offs to be made is that elimination of entrainment and impingement by converting once-through power plants to closed-cycle cooling would be extremely expensive. In 1992 the estimated capital cost of converting all once-through plants to closed-cycle was $23 billion to $24 billion[30]. The extra electrical energy required to operate cooling towers and the reduced output from less efficient operation was estimated to cost an additional $13 billion to $24 billion[31], bringing the total cost to $36 billion to $48 billion. The prudence of the expenditure of this magnitude to eliminate entrainment and impingement losses when population level effects are not detectable is questionable.

Environmental impacts of other sorts are also a trade-off when once-through cooling is replaced by closed cycle. These impacts include destruction of vegetation and terrestrial habitat, noise, visual impacts, additional fuel use, increased air emissions, and construction-period impacts for any type of cooling tower. In addition, aerosol and saline drift, plumes, fogging, icing, discharge of chemicals and biocides, and evaporative water loss may be issues for wet towers.

Given the greater degree of certainty of assessment of effects that can be achieved in 2001 than was possible in 1972, it would seem logical that the degree of conservatism of regulatory approach could be reduced. In 1977, Van Winkle described the state of knowledge of assessing population-level power plant impacts from the viewpoint of an optimist, a pessimist, and a realist[32]. At that time, four aspects of population assessments needed improvement: estimating abundance, production, and mortality rates; monitoring programs and data analysis; compensation and stock-recruitment relationships; and use of population models. All four of these aspects have been explored diligently in the last 24 years, and many significant advances have been made. Although Van Winkle's optimist, who viewed these aspects as completely resolvable, has not been proven totally correct, his realist, who envisioned that significant improvements were possible, was probably not far off.

Have the reductions in uncertainty achieved over the last quarter-century been translated into reductions in conservatism in regulatory philosophy? Two east-coast states provide an interesting contrast in regulatory viewpoint. The state of Maryland appears to have adopted the "realist" viewpoint that population assessments remain uncertain, but data collected to date have shown that healthy populations and once-through cooling systems are not mutually exclusive. Maryland's regulations specifically exempt intakes of less than 10 million gallons per day (mgd)[33], presumably because intakes of this size would not be able to significantly harm the resident populations. Maryland also has a set formula for determining when costs and benefits of alternative technologies exceed the "wholly disproportionate" test.

The Maryland approach is in sharp contrast to that of the state of New York, which decidedly takes the pessimistic view. In a recent decision on best available technology for the proposed 1080 MW combined-cycle Athens Generating Station, the NYSDEC commissioner ruled that dry cooling was the best available technology for the plant, over the hearing examiner's recommendation that a hybrid wet-dry cooling tower, with wedge-wire screened intakes, and a fabric filter curtain would be sufficient. The commissioner found that the 4.2 mgd average flow with the hybrid towers and wedge-wire screens would kill 24,500 young-of-year American shad (0.2% of the population) and 1.8 million river herring (0.3% of the population), and would be unacceptable. In his view the hearing record did not support the additional application of a fabric filter curtain. Dry cooling would withdraw only 0.18 mgd and kill an estimated 1,000 young-of-year American shad and 76,500 young-of-year river herring annually. In the eyes of the commissioner, the incremental cost of $39 million for the dry cooling system over an assumed 20-year life of the plant was not "wholly disproportionate" to the environmental benefits to be gained[34]. The decision did not state what the benefits to be gained were, other than impact to aquatic organisms would be minimized. According to the decision, the applicant has the burden of proof to demonstrate that costs and benefits are disproportionate.

One might expect, given the highly conservative nature of the Athens decision, that New York had much more stringent regulations for cooling water intakes, but, in fact, the New York regulations simply parrot the language of 316(b). The state has not issued any formal guidance or regulations that support such a conservative interpretation. Like the federal government, New York State has not formally defined "adverse environmental impact." However, in comments to USEPA, one New York regulator proposed that adverse environmental impact was "any harmful, unfavorable, detrimental or injurious effect on *individual* (emphasis added) organisms of fish, wildlife or shellfish or their eggs or larvae; or the water, land or air resources of the U.S…..; or on human health, welfare, or safety; or on the human enjoyment of those resources"[35].

The reason given for proposing this simplistic definition is to avoid "analysis paralysis" that may result from a more complex standard. The New York regulator cited the Hudson River case as a prime example of this paralysis. After millions of dollars have been spent on environmental research for more than 25 years, "the state agency, regulated parties, and citizen conservation groups *still disagree with the interpretation,* despite probably the best data set on the planet, full agreement on sampling design, data collection, certain analysis techniques, and many aspects of modeling." This "paralysis" is used as an argument that a population-based standard is unworkable, yet the reality is that the paralysis occurs because there is no standard against which the data and analyses can be evaluated. If either USEPA or New York had adopted a workable population-based standard for adverse environmental impact, then it would be clear from the "best data set on the planet" whether the standard had been met. Certainly, if the 25+ years of Hudson River data are not sufficient to assess whether adverse environmental impact has occurred, then it is unlikely that any data set will prove adequate for the task.

Does a standard such as that being used in New York arise from a need to be conservative in the face of uncertainty, or from other considerations? In objecting to USEPA's proposal for cost-benefit analysis, the New York regulator stated, "EPA has no right to allocate State public trust resources to be killed in this manner." Clearly, New York has decided there are legitimate and illegitimate sources of fish mortality, and power plants fall into the latter category. Recreational and commercial fishing both are industries that derive income from the taking of fish, either by intent (legal sizes of target species) or by accident through the by-catch. However, New York's position is that these industries differ from power generation in that they have a historical and societal right to take fish. By categorizing industry-based mortality into legitimate and illegitimate sources, New York has no need to develop a logical, science-based approach to definition of adverse environmental impact.

After a quarter century of case-by-case decisions on 316(b) requirements, we still have plants using both once-through and closed-cycle cooling. Although we can't determine what would have happened had the plants with closed-cycle cooling not installed that technology, we can see, from those that have once-through systems, that local fish populations have not been decimated by entrainment or impingement[36]. There are no documented instances of populations being driven to the brink of collapse by power plant cooling systems. For systems that have been studied for long time periods, there is empirical evidence that, even with non-trivial levels of direct effects (conditional mortality rates on the order of 10% or more), fish populations continue to remain healthy[36,37,38]. If we have learned nothing else from the millions of dollars spent on studies and monitoring, we should have learned that there is not a one-size-fits-all solution to the best available technology requirement. Can we afford to be overly conservative on the cooling water intake issue when other environmental threats that appear more serious will also require resources to resolve?

We have now made it to the twenty-first century, so the accuracy of the quotes at the beginning of this paper is easily assessed. So far there have been no reports of any major rivers reaching the boiling point or entirely evaporating away as a result of heated discharges. In contrast to the 1.5 million megawatt demand envisioned for the end of the century, in 1999 the actual generation capacity in the United States was only 785,990 megawatts, about 50% of the prediction. In a similar vein, the dire prediction for the Hudson River striped bass population subject to entrainment and impingement has also not come to pass. It would seem logical that regulatory agencies would recognize the advances made in population assessments, and the empirical demonstrations of still healthy fish populations and communities, and adjust the conservatism of regulatory policies accordingly.
